# Experiencing the impossible and creativity: a targeted literature review

**DOI:** 10.7717/peerj.13755

**Published:** 2022-07-20

**Authors:** Richard Wiseman, Caroline Watt

**Affiliations:** 1 Department of Psychology, University of Hertfordshire, Hatfield, Hertfordshire, United Kingdom; 2 School of Philosophy, Psychology and Language Sciences, University of Edinburgh, Edinburgh, Midlothian, United Kingdom

**Keywords:** Magic, Impossible, Dreaming, Science fiction, Paranormal, Conjuring, Play

## Abstract

Previous work suggests that unexpected and surprising experiences (*e.g*., living in another culture or looking at surreal images) promotes creative thinking. This targeted literature review examines whether the inherent cognitive disruption associated with experiencing the seemingly impossible has a similar effect. Correlational and experimental research across six domains (entertainment magic, fantasy play, virtual reality and computer gaming, dreaming, science fiction/fantasy, and anomalous experiences) provided consistent support for the hypothesis. In addition, anecdotal evidence illustrated the possible impact that the creative output associated with each of these areas may have had on technology, science, and the arts. It is argued that impossible experiences are an important driver of creative thinking, thus accounting for reports of such experiences across the lifespan and throughout history. The theoretical and practical implications of this work are discussed, along with recommendations for future research.

## Introduction

*Alice laughed: “There’s no use trying,” she said; “one can’t believe impossible things.”*

*“I daresay you haven’t had much practice,” said the Queen. “When I was younger, I always did it for half an hour a day. Why, sometimes I’ve believed as many as six impossible things before breakfast.”*

*Through The Looking Glass* by Lewis Carroll.

People experience the seemingly impossible when they encounter an event that they believe cannot occur. Such experiences take place in a diverse range of contexts, including those in which the event is believed to be genuine (*e.g*., when they have a paranormal or exceptional experience) and those that are thought to be illusory (*e.g*., when they watch a conjuring show, look at an optical illusion, have a bizarre dream, become absorbed in a magical story, or engage in fantasy play). A large body of evidence suggests that these experiences occur across the lifespan and have been reported throughout history. For example, almost all children engage in fantasy play (Woolley, Bunce & Boerger, 2020), bizarre dreams have been reported for millennia (Bulkeley, 2016), conjurors have entertained audiences for hundreds of years (Lamont & Steinmeyer, 2018), and almost every culture has developed some form of magical storytelling (Warner, 2014).

Many people trivialise such experiences and frequently see them simply as a source of entertainment or fun (*e.g*., play, magic tricks, and storytelling). In general, they have not attracted a significant amount of attention from psychologists and much of the research that has been conducted has focused on domain specific issues, such as identifying the cognitive biases that underpin magic tricks (*e.g*., Kuhn, 2019), examining how children distinguish reality from illusion (*e.g*., Woolley & Ghossainy, 2013), or investigating the nature of bizarre dreams (*e.g*., Bulkeley, 2016). In contrast, little work has attempted to develop a more general understanding of such experiences by identifying commonalities across these diverse domains. This is unfortunate, in part, because this broader approach has the potential to provide valuable insights into several issues, including why humans have evolved to experience the impossible, and the effect that such experiences have on cognition, motivation and emotion. The current article illustrates the benefits of this general approach by examining one such issue, namely the degree to which experiencing the impossible boosts creative thinking.

Researchers have long debated the definition of creativity (*e.g*. Sternberg & Lubart, 1999; Runco & Jaeger, 2012; Walia, 2019), with one widely accepted approach involving two key components: originality and effectiveness. For an idea to be seen as original it must be novel or unusual, whereas to be effective it needs to be useful or relevant to the task at hand. Measuring creativity is equally contentious, with researchers employing three main types of instrument. First, many studies employ tests, such as Guilford (1967) Alternative Uses Test (AUT: wherein participants generate multiple uses for everyday objects and these responses are then scored for Fluency [number of ideas], Originality [novelty of ideas] and Flexibility [number of conceptual categories generated]) and the Remote Associations Test (RAT: wherein participants try to generate a word that is related to three other words). Second, some studies involve more real-world tasks, such as asking participants to design a new product, and then having expert judges score the responses for originality, feasibility, *etc*. Finally, some researchers adopt a trait approach, asking participants to complete questionnaires that explicitly refer to creativity (*e.g*., the Adjective Checklist, Gough & Heilbrun, 1965) or indirect measures that correlate with creative thinking (*e.g*., the ‘Big Five’ trait of Openness to Experience, tolerance to uncertainty or dogmatism).

A large body of work has examined the procedures and techniques that influence creativity (for a review, see Vernon, Hocking & Tyler, 2016), with two separate but related strands of research examining impact of experiences that disrupt habitual and stereotypical thinking. One strand of this work has investigated the impact on creativity of unusual but relatively normal experiences, such as travelling to other cultures and living abroad (Leung et al., 2008; de Bloom et al., 2014), generating non-stereotypical social categories (*e.g*., Gocłowska, Crisp & Labuschagne, 2013), viewing incongruous images (*e.g*., Gocłowska et al., 2017), and engaging in an unusual series of actions (*e.g*., Ritter et al., 2012). Findings across these studies support the notion that these sorts of events promote creative thinking.

A second strand of work has explored the relationship between awe-inspiring phenomena and creativity. People experience a sense of awe when they encounter stimuli that are both vast and challenge their understanding of the world (Keltner & Haidt, 2003), such as exceptional natural phenomena, highly talented individuals, or extraordinary art. Correlational studies have reported positive relationships between the propensity to experience awe and several measures of creative thinking (*e.g*., Shiota, Keltner & Mossman, 2007; Silvia et al., 2015; Zhang et al., 2021), and experimental studies suggest that that awe-inspiring experiences enhance creativity (*e.g*., Shiota, Keltner & Mossman, 2007; Chirico et al., 2018b).

Although the cognitive mechanisms that underpin these approaches are somewhat unclear, most of the theoretical work in the area has focused on the notion of schema disruption. The term ‘schema’ is used to describe the mental models that represent the organization of information about a certain topic. A large amount of work has examined how these models influence thinking, help to shape expectations and attitudes, and guide behaviour (*e.g*., McVee, Dunsmore & Gavelek, 2005). During problem solving, individuals who rely on existing schemata to generate potential solutions are likely to produce conventional and stereotypical solutions (Marshall, 2012). According to some researchers, unusual experiences disrupt people’s schemata by modifying existing models and generating new categories, and thus result in more creative thinking (*e.g*., Gocłowska, Crisp & Labuschagne, 2013; Gocłowska et al., 2017).

In line with this work, several researchers have noted that the inherent schema disruption associated with impossible experiences is likely to boost creative thinking (*e.g*., Ritter et al., 2012; Li, 2020; Wiseman, Wiles & Watt, 2021). Indeed, some psychologists have argued that such experiences may be especially effective at enhancing creativity because they appear to violate people’s fundamental assumptions about themselves and the world (*e.g*., Singer & Singer, 1990; Rosengren, Johnson & Harris, 2000; Subbotsky, 2010; Bulkeley, 2016). Researchers working in a diverse range of domains have carried out studies and collected anecdotal evidence relevant to this idea. Unfortunately, this work is often unknown to researchers outside each of these domains. This article brings this diverse body of work together and presents a synthesising narrative review.

## Search strategy

English-language psychology-based literature relating to impossible experiences and creativity was identified by first entering the search term ‘creativity’ coupled with ‘impossible’, ‘fantasy’, ‘magic’ into academic databases (including Google Scholar, Ovid Medline, and Scopus). The resulting references were sorted for relevance, and the titles and abstracts of the top papers were searched to identify work that involved (i) participants having read about, watched, or experienced something impossible and (ii) a quantitative or qualitative measure of creativity. These papers and books were then located, and additional material identified by examining the items referenced in these works. All searches were undertaken in March 2022. The first author sorted the resulting literature into different categories, and these were examined by the second author, with any discrepancies being resolved by discussion. The final grouping consisted of six areas: Entertainment magic, dreaming, virtual reality and computer gaming, fantastical play, science fiction/fantasy and anomalous experiences. The papers obtained in each of these categories are briefly described in the Appendix.

## Review

### Entertainment magic

Magicians appear to achieve the impossible by making objects seem to disappear, transform, levitate, etc. During a magic trick, people experience a series of events that they believe cannot happen. This experience does not involve any suspension of disbelief (Lamont, 2010; Lamont, 2017; Leddington, 2016). For example, when a magician appears to levitate, the spectator must not be able to figure out the method, whereas during a play the audience might be able to see the wires supporting the performer but are required to imagine that the cables are not present. As such, it seems likely that watching a magic trick will involve a considerable amount of cognitive disruption and, according to schema disruption theory, increase creativity.

Several illusionists have argued that watching magic results in a more expansive mindset. For example, illusionist David Copperfield often describes how his shows aim to inspire people to believe that the seemingly impossible might be possible (Morehart, 2021) and magician Marco Tempest frames conjuring tricks as a way of ‘prototyping the future’ (Royal Society of the Arts, 2013). In line with this notion, many famous magicians have produced highly innovative solutions to real world problems, including Nevil Maskelyne’s ground-breaking research into wireless telegraphy, Georges Méliè’s pioneering work in early cinema (Barnouw, 1981), and Jean-Eugène Robert-Houdin’s creation of a prototype smart home in the 1850s (Smith & Lewi, 2008).

Although several studies have investigated the divergent thinking that underpins many illusions (*e.g*., Danek et al., 2014; Hedne, Norman & Metcalfe, 2016), only a relatively small amount of work has explored whether experiencing entertainment magic can enhance creativity. Silvia et al. (2022) had adult participants complete a self-report measure that reflected the degree to which they dislike watching magic, and three questionnaires indirectly related to creativity (Openness to Experience, Brief Dogmatism Scale, and Intolerance of Uncertainty Scale). Participants who liked magic tended to be more open, less dogmatic and have a higher tolerance of uncertainty. Wiseman, Wiles & Watt (2021) had 10-year-old children complete the AUT both before and after having taken part in either a magic-based (learning how to perform a magic trick) or art-based (learning how to make a perspective drawing) activity. Compared to the art-based activity, the magic-based activity resulted in significantly higher Fluency and Originality scores.

In two studies, Haritaipan, Saijo & Mougenot (2018a, 2018b) informed design students about different kinds of magical illusions and then had them create novel designs for everyday objects. Judges then blind rated these ideas for Fluency, Flexibility, and Originality. Overall, the students obtained higher Flexibility and Originality scores than a control group that hadn’t been informed about the illusions. In Li (2020), design students completed the AUT, participated in a magic-based intervention (in which they saw a magic trick, discovered how it was achieved and performed the trick for each other), were interviewed about their experiences and again completed the AUT. Participants described how the intervention boosted their creative thinking and their post-intervention Fluency scores were significantly higher than their initial scores.

In short, correlational work shows that creativity is positively related to liking magic, and experimental studies suggest that experiencing magic boosts creative thinking. This latter strand of work has yet to determine whether this effect is due to participants simply watching magic or also discovering the methods that underpin the illusions.

### Fantasy play

Children frequently simulate seemingly impossible events during play, including pretending to have magical powers, imagining that their bedroom is a fairy castle, or conjuring up mythical entities such as gnomes and elves (*e.g*., Woolley, 1997). Fantasy play involves the mental creation of scenes, objects, characters, and worlds that are impossible. Such activities are highly likely to result in schema disruption and thus would be expected to enhance creativity.

Anecdotally, many highly creative individuals have described creating complex magical worlds when they were children, including both famous writers (*e.g*., the Bronte Sisters, Lewis Carroll, Robert Louis Stevenson, and J. R. R. Tolkien), and MacArthur Fellows working in both the arts and sciences (Root-Bernstein & Root-Bernstein, 2006; Root-Bernstein, 2014).

Several correlational studies have explored the relationship between fantasy play and creative thinking (for a review, see Woolley, Bunce & Boerger, 2020). For example, Russ & Grossman-McKee (1990) reported a positive correlation between the degree to which children incorporated fantastical elements into their play and AUT Flexibility scores. Using a similar approach, Russ, Robins & Christiano (1999) reported that the degree to which children engaged in fantasy play was positively related to their AUT Fluency and Flexibility scores over a 4-year period. Similarly, Taylor et al. (2020) noted that children who imagined highly detailed impossible worlds during play also obtained significantly higher scores on several socially-oriented creativity tests (*e.g*., producing creative endings to stories and drawing a pretend person). Most recently, Bunce & Woolley (2021) found that children’s level of fantasy play was positively associated with measures of verbal (naming as many red objects as possible) and physical (finding different ways of moving between two lines marked on the floor) creativity, but not artistic creativity (draw a picture of a real and pretend person).

Only one study has explored the causal link between fantasy play and creativity. Hoffmann & Russ (2016) had children complete the AUT both before and after a 3-week storytelling course. During the course, the children were encouraged to create several stories, with the material slowly encouraging more fantastical and magical elements over time. The children’s post-course AUT Fluency and Originality scores were significantly higher than their initial scores.

In sum, a relatively large body of work suggests that engaging in fantastical and magical play is related to creativity. However, most of this work is correlational in nature, and thus it is difficult to identify the direction of causality.

### Science fiction/fantasy

Science fiction and fantasy narratives transport people into alternative worlds in which events that are currently impossible appear possible. Schema disruption theory predicts that engaging with such narratives will boost creative thinking. Anecdotal evidence suggests that those creating such narratives envision seemingly impossible technology long before it becomes a reality. For example, Jules Verne described humans going to the Moon over a century before the Apollo missions, Edward Bellamy predicted the use of credit cards in the 1880s, and Arthur C. Clarke envisaged the emergence of tablet computers. On some occasions this work has helped inspire people to make seemingly impossible technologies and achievements possible. For example, Scott & Jurek (2014) have described how science fiction books, comics, and radio shows about going to the Moon played a key role in persuading the public and policymakers to support the NASA lunar program.

Several correlational studies suggest that reading science fiction/fantasy stories is positively related to openness to experience (*e.g*., Fong, Mullin & Mar, 2013; Stern et al., 2019). In addition, three experimental studies have examined the degree to which watching fantasy-based stories promotes creativity.

Subbotsky, Hysted & Jones (2010) had 4- and 6-year-old children watch a film that either featured ‘magical’ events (*e.g*., talking animals or magicians performing the impossible) or involved the same characters performing non-magical actions. Participants completed Torrance’s Thinking Creatively in Action and Movement Test both before and after watching the films. These tests involve a variety of tasks, including moving across a room in different ways, drawing pictures of non-existent toys and fruit, and coming up with different methods of putting plastic cups into a bin. Compared to the non-magical film, the magical film resulted in significantly higher scores on three dimensions (Fluency, Originality, and Imagination). These findings were replicated in a second experiment involving children aged 6 to 8 years old.

Similarly, Black & Barnes (2021) had groups of adult participants watch either a 40-min science fiction television programme, a more realistic television show (a crime drama or western) or no film at all. Participants then completed the AUT and Green & Brock’s (2000) Transportation Scale, which measure levels of absorption in narratives. For those watching the science fiction film, greater levels of transportation were associated with higher AUT Fluency scores. This was not the case for those watching the realistic television programme or the control group.

Finally, Lin (2014) examined the effect of exposure to science-fiction on a design task. Junior high school students either watched a science fiction film or were taught traditional problem-solving techniques, completed the Creativity Assessment Packet (a test of creative thinking similar to the AUT) and designed robotic cross-country vehicles. The students who had watched the film obtained higher creativity scores than those in the control group, but did not produce more creative vehicle designs.

A related strand of research has explored whether exposure to science fiction makes people more open to radically alternative future possibilities. Shahghasemi & Khaleghipoor (2021) interviewed 6-to 12-year-old children about the time spent watching fantasy-oriented animations and their ideas about how to change the world. The children who didn’t watch fantasy animations tended to focus on more realistic options (*e.g*., covering the world in grass and trees), whilst those who preferred fantasy animations tended to adopt a more expansive approach, producing both reality-based ideas and impossible options that might be possible in the future (*e.g*., developing magical powers). Similarly, working with an adult cohort, Black, Capps & Barnes (2018) found a positive correlation between the degree to which people read or watched science fiction/fantasy and their belief that impossible phenomena (*e.g*., telepathic communication, time travel, and humans being able to fly) might be possible in the future.

In summary, several experimental studies show that engaging with science fiction/fantasy stories promotes divergent thinking. Additional work suggests that such narratives can also encourage people to believe that impossible phenomena will be possible in the future.

### Virtual reality and computer gaming

Virtual reality immerses people in computer-generated environments, allowing them to experience fantastical worlds and impossible events (*e.g*., Bakk, 2020; Velasco et al., 2021). This experience is likely to disrupt existing schemata so would be expected to enhance creativity. A small amount of work has tested this notion. One study has examined whether such experiences enhance creativity. Ritter et al. (2012) placed students into a virtual environment and had them experience a 3-min walk through a university cafeteria. Some participants saw impossible events (*e.g*., objects increasing in size or defying gravity), others experienced ‘normal’ versions of the same events whilst a third group watched a film depicting the impossible environment. Participants then completed a version of the AUT, and those who had experienced the impossible environment obtained higher AUT Flexibility scores than those in the other two groups. Although the higher scores associated with experiencing the impossible environment is in line with schema disruption theory, the lower scores associated with observing the impossible run contrary to other studies examining the impact of exposure to impossible narratives.

Some computer games immerse players in fantastical worlds and allow them to perform impossible feats. Although several studies have assessed the impact of computer games on creativity (for a review, see Rahimi & Shute, 2021), no studies have explicitly explored this dimension. However, some work has examined the relationship between playing Minecraft (in which people can create both realistic and fantasy worlds) and creative thinking. Minecraft players report that the game helps to boost creativity (*e.g*., Sáez-López et al., 2015) but experimental work has found mixed results. One small-scale study found no differences in participants’ Originality, Flexibility or Fluency scores on the Torrance Tests of Creative Thinking after playing Minecraft, a puzzle game, or a shooter game (Moffat, Crombie & Shabalina, 2017). In a larger study, Blanco-Herrera, Gentile & Rokkum (2019) reported that participants’ scores on a drawing-based creativity task (in which they drew a creature from another world, with less human-like aliens obtaining higher scores) and AUT Fluency were significantly greater after playing Minecraft compared to a racing game or watching television. There were no differences between the groups for AUT Originality and RAT scores.

### Impossible dreams

Dreams often contain bizarre and impossible imagery. For example, around half of the population reports having flown in a dream and having experienced an impossible visitation, such as meeting a mythical creature or a deceased person (Bulkeley, 2016). Similarly, during lucid dreaming, people frequently have experiences that are impossible in everyday life, such as performing magic, talking with animals, and travelling through time (Stumbrys et al., 2014). Experiencing and recalling this type of dream content is likely to disrupt existing schemata and thus would be expected to enhance creativity. Anecdotally, there are many reports of such dreams inspiring scientists to come up with innovative theories (*e.g*., Dmitri Mendeleev’s creation of the periodic table and August Kekulé’s discovery of the structure of benzene) and writers to develop pioneering narratives (*e.g*., Mary Shelley’s ‘Frankenstein’ and Robert Louis Stevenson’s ‘The Strange Case of Dr Jekyll and Mr Hyde’). Bizarre dreams have also inspired the work of many visual artists, including, most famously, surrealists such as Salvador Dali and Max Ernst.

Although a large body of work has examined the positive relationship between dreaming and creativity (*e.g*., Schredl & Erlacher, 2007; Barrett, 2017), only a relatively small number of studies have focused on dreams that contain impossible and bizarre content. Domino (1976) classified schoolchildren as creative or non-creative based on their AUT and RAT scores, along with teacher ratings for ‘originality, adaptiveness to reality and elaboration of original insight’. The pupils were then asked to keep a dream diary for 2 weeks. Compared to the non-creative students, the dreams described by the creative students obtained consistently higher blind ratings on scales reflecting impossibility, bizarreness, and uncanniness. Sladeczek & Domino (1985) carried out a similar study with adult participants from ‘creative’ (*e.g*., architects, musicians, sculptors) and ‘non-creative’ (*e.g*., administration and accountancy) professions. The groups were matched on age, sex, and education. Once again, compared to the dreams described by non-creative participants, the dreams from the creative participants were blind rated as significantly more implausible, unrealistic, and bizarre.

Several studies have revealed a strong and positive correlation between frequency of lucid dreaming and scores on self-report creativity measures (*e.g*., Blagrove & Hartnell, 2000; Zink & Pietrowsky, 2013). Tholey (1989) demonstrated that lucid dreamers could be instructed to dream about meeting individuals who only existed in their dreams, and found that these imaginary characters helped with simple creativity tasks such as finding rhyming words. However, further work by Stumbrys & Daniels (2010) suggests that the solutions provided by these ‘dream characters’ were no more original than answers provided by a group of non-lucid dreamers.

In short, correlational studies suggest that having impossible dreams is related to creativity. However, the difficulties associated with inducing such dreams means that no research has examined the direction of causality.

### Anomalous experiences

People report a range of anomalous and paranormal experiences, including instances of apparent telepathy and precognitive dreaming, near-death and out-of-body experiences, and hallucinations (French & Stone, 2013; Watt, 2016). Although most individuals interpret such experiences as genuine (*i.e*., that they reflect the existence of paranormal or extraordinary phenomena) some do view them as illusory (*i.e*., that they are somehow imagined or unreal). Either way, such experiences frequently involve a radical challenge to a person’s existing understanding of the world and thus considerable disruption to their schemata and would be expected to boost creativity. Anecdotally, there are many reports of several well-known scientists having such experiences. For example, Alfred Russel Wallace (who independently came up with the theory of evolution) regularly experienced apparent séance phenomena, Hans Berger (who created the first EEG machine) had several alleged telepathic experiences, and William Crookes (who discovered the element thallium) conducted several experiments that supported the existence of psychic ability. Similarly, within the arts, surrealist writer André Breton experienced mediumistic phenomena, Victor Hugo practised automatic writing and drawing, Arthur Conan Doyle regularly attended séances, Upton Sinclair conducted telepathy experiments with his wife, and British Poet Laureate Ted Hughes frequently attended Ouija sessions (Cardeña, Iribas & Reijman, 2012).

A large amount of correlational work has examined the relationship between alleged paranormal experiences and creativity (for a review, see Holt, 2012). For example, several studies have found a positive correlation between reports of anomalous experiences on schizotypy questionnaires, and both RAT and AUT scores (*e.g*., Batey & Furnham, 2008; Schuldberg, 2000). Similarly, other work has found positive correlations between reports of anomalous experiences and several measures of creativity, including the Torrance Creative Motivation Inventory (Thalbourne & Delin, 1994) and the degree to which creativity is important in people’s lives (Kennedy & Kanthamani, 1995). Taking a somewhat different approach, Ayers, Beaton & Hunt (1999) found that those in creative professions (*e.g*., actors and artists) report more anomalous experiences.

It has proved challenging to conduct studies examining the causal role of anomalous experiences due to the difficulties associated with inducing such experiences. Mixed results have been obtained by researchers attempting to create anomalous experiences *via* alcohol, hypnosis, and psychedelic substances (Holt, 2012). In contrast, work on sensory isolation has proved more promising. For example, Norlander, Kjellgren & Archer (2002–2003) had participants either spend time in a floatation tank or rest in a darkened room, and then write an essay based around four target words. Those who had been in the tank reported significantly more anomalous experiences and produced more original essays. Related work suggested that the floatation experience also resulted in higher Originality (but not Fluency) scores on a version of the AUT (Norlander, Bergman & Archer, 1998). Taking a different approach, Braud et al. (2007, reported in Braud 2012) had participants imagine reliving a previous anomalous experience or an everyday experience, and then describe their thoughts and feelings. Qualitative analyses suggested that whereas reliving the typical experience was associated with a ‘business as usual’ approach, the reliving of the anomalous experience was associated with increased openness, creativity, playfulness, and curiosity.

In sum, correlational studies suggest a positive relationship between anomalous experiences and creativity. The difficulties associated with inducing such experiences have restricted experimental work in this area, although some research using sensory isolation and the imaginal reliving of anomalous experiences has yielded promising results.

## Discussion

Researchers have speculated that the inherent cognitive disruption associated with experiencing the impossible may enhance creative thinking. Studies examining this notion have been carried out within six diverse domains. This work has employed a variety of designs (including correlational studies and experimental research) and used several types of creativity measures (including tests of divergent thinking and real-world problem solving). Correlational work in all the domains showed that experiencing the impossible was positively related to creative thinking. In addition, experimental work in several domains suggested that such experiences played a causal role in enhancing creativity (entertainment magic, magical play, anomalous experiences, science fiction/fantasy, virtual reality). Anecdotal evidence also suggested that the creative outputs associated with each area may have had an impact on many aspects of society, including technology, science, and the arts.

These findings have several theoretical implications. First, they are consistent with previous work suggesting that both unusual and awe-inspiring experiences enhance creative thinking. Second, they have the potential to expand our understanding of problem solving and prospection (the generation and evaluation of psychological representations of possible futures). Research into both areas has traditionally focused on reality-based solutions and possibilities. However, the work reviewed in this article suggests that the ability to enter an imaginary world in which anything is possible helps people to generate highly creative solutions and to consider radically different futures. As such, impossible experiences may play a vitally important, but hitherto largely unrecognised, role in both problem solving and prospection. Seen from this perspective, such experiences are not just sources of entertainment and fun, but also possess considerable psychological importance. This may help to account for the prevalence of such experiences across the lifespan and throughout history. This reframing also gives rise to a novel way of explaining why some people endorse impossible phenomena based on very little evidence (*e.g*., some allegedly paranormal phenomena and conspiracy theories), with such erroneous beliefs possibly being the price paid for being open to other seemingly impossible ideas that eventually prove to be highly valuable. Conceptually, this reflects recent work exploring the adaptive value of illusory experiences, wherein, for example, pareidolia (mistakenly seeing faces in non-facial stimuli) is seen as the result of a facial recognition system that tolerates false positives to avoid the high costs associated with false negatives (Palmer & Clifford, 2020).

In addition, our review also highlights several possible avenues for future research.

First, much of the work reviewed here was correlational in nature so it isn’t possible to determine the direction of causality. It is possible, for example, that impossible experiences cause people to be more creative and/or more creative people are attracted to impossible experiences. This issue can only be resolved by building on the relatively small number of studies that have adopted a more experimental approach. Future research could employ some of the many techniques reviewed here to generate impossible experiences, including having people watch magic tricks, become immersed in virtual reality, read magical narratives, and engage in fantasy play. Additional work could explore how to sharpen and improve these techniques to ensure that they are maximally effective. Part of this research could involve building on domain-specific literature on how to create impossible and extraordinary experiences within magic (*e.g*., Ortiz, 1994; Ozono et al., 2021), virtual reality (*e.g*., Chirico et al., 2018a), computer gaming (*e.g*., Kumari, Deterding & Kuhn, 2018), and design (*e.g*., Tognazzini, 1993). Also, given that many of these procedures involve the willing suspension of disbelief, it may prove especially productive to explore how to promote levels of absorption and transportation. This line of inquiry could examine the individual and situational factors that promote absorption during play and while individuals engage with sci-fi/fantasy narratives. Interestingly, some researchers have argued that magic tricks do not require the suspension of disbelief (Lamont, 2010; Lamont, 2017; Leddington, 2016) and so they may prove an especially useful way of providing a wide range of people with impossible experiences. Additional work could explore the efficacy of procedures designed to promote such experiences in more challenging domains, especially those associated with dreaming (Horowitz et al., 2020), lucid dreaming (Stumbrys et al., 2012) and anomalous experiences (Cardeña & Winkelman, 2011). This work could also examine the longevity of effects. Most of the experimental research presented here involved testing participants immediately after having an impossible experience, and future work could examine how long these effects last and how this time period could be maximised. Finally, this research could also adopt a muti-factor approach and investigate the mechanisms underlying the effects including, for instance, the role played by emotion, suggestion, and absorption.

Second, researchers could examine whether the same types of effects emerge when people experience the impossible in other contexts, including when they observe optical illusions, or visit immersive attractions and theme parks. Whereas most of the impossible experiences discussed in this review are considered illusory, future work could also focus on experiences that are widely perceived to be genuine. This could, for example, include learning about individuals who achieved something that was widely considered to be impossible (*e.g*., the Apollo Moon landings or Roger Bannister running a mile in under 4 min), encountering technology that is so extraordinary that it appears magical, or experiencing unbelievable scientific demonstrations or natural phenomena.

Third, additional work could also develop and test the use of interventions in a range of applied settings, including helping those with mental health issues to imagine a more positive future, raising the career aspirations of children, assisting designers to develop highly innovative products, and encouraging the public and politicians to imagine a more sustainable and just world. Existing work in many of these areas seems promising. For example, some storytellers are studying whether placing children in a magical environment enhances their creativity (Wyse et al., 2020), educational practitioners are examining how superhero play and other magic-related activities promote creativity in the classroom using (Pollock, 2015; Grimmer, 2019), magicians are investigating the impact of incorporating magical experiences in everyday life (Tomatis & Buscema, 2020), researchers are exploring how awe-inspiring experiences may help to alleviate depression (Chirico & Gaggioli, 2021), and designers are creating science fiction stories as a way of helping them to predict future trends and needs (Johnson, 2011).

Fourth, at a more conceptual level, this work demonstrates that it’s possible to identify commonalities across a diverse range of seeming impossible experiences. This raises the possibility of building a more general psychology of such experiences, with additional work exploring other potential commonalities, including optimism, hope, wonder, surprise, attention, memory, self-identity, and curiosity.

## Conclusions

A considerable amount of research across a diverse range of contexts suggests that experiencing the impossible promotes creative and expansive thinking. In addition, anecdotal evidence suggests that such experiences have made a significant impact on many aspects of society. Future work in this area could involve creating and sharpening new and existing interventions, and applying these interventions across a range of real-world settings. In addition, this review illustrates the potential to build a more general theory of impossible experiences. It is hoped that this article will encourage other researchers to study these intriguing and important experiences, and to follow Lewis Carroll’s timeless advice regarding the benefits of believing six impossible things before breakfast.

## Figures and Tables

**Appendix:** Characteristics of studies included in the review.

Entertainment magic		
**Author(s) & date**	**Study participants, variables, and procedure**	**Outcome**
Silvia et al. (2022)	Adults completed Loathing of Legerdemain scale (LOL), Openness to Experience, Brief Dogmatism Scale, Intolerance of Uncertainty Scale.	Negative correlations between LOL and other scales.
Wiseman, Wiles & Watt (2021)	Children taught to perform magic trick vs art lesson. Completed AUT Fluency and Originality pre and post intervention.	Magic increased Fluency and Originality.
Haritaipan, Saijo & Mougenot (2018a)	Design students shown written descriptions of magic effects, videos of effects or nothing. Created chair designs rated for Fluency, Flexibility, and Originality.	Magic descriptions and videos increased Originality.
Haritaipan, Saijo & Mougenot (2018b)	Design students shown written descriptions of magic effects, effects plus methods, or nothing. Created mug designs rated for Fluency, Flexibility, and Originality.	Both magic groups obtained higher Originality and Flexibility.
Li (2020)	Students taught to perform magic tricks. Completed AUT Fluency pre and post intervention.	Higher Fluency post training
Fantasy play		
Russ & Grossman-McKee (1990)	Children completed Affect in Play Scale (Fantasy sub-scale) and AUT Flexibility.	Positive correlation between measures.
Russ, Robins & Christiano (1999)	Children completed Affect in Play Scale (Fantasy sub-scale) and AUT Flexibility and Fluency.	Positive correlations between measures.
Taylor et al. (2020)	Children interviewed about whether they created paracosms, and completed social creativity tests (story completion and making friends [Study 2 only]), and non-social tests (AUT, collage completion, draw-a-pretend-person, consequences).	Paracosms associated with higher story completion (Study 1 & 2), draw-a-person (Study 1) and making friends (Study 2).
Bunce & Woolley (2021)	Children scored on fantasy play (*via* Imaginative Play Predisposition, Imaginary Companion, Impersonation interviews), and completed verbal (name red objects), physical (move between two lines), and artistic (draw real, pretend and impossible person) creativity tasks.	Fantasy play positively correlated with verbal and physical tasks.
Hoffmann & Russ (2016)	Children engaged in fantasy-based storytelling sessions vs art-based control. Completed pre and post AUT Fluency and Originality, and Affect in Play Scale (Imagination sub-scale).	Storytelling increased Fluency, Originality, and Imagination.
**Science fiction and fantasy**		
Fong, Mullin & Mar (2013)	Students completed Author Recognition Task-Revised (domestic fiction, romance, sci-fi/fantasy, and suspense/thriller) and Big Five Inventory.	Sci-fi/fantasy positively correlated with openness.
Stern et al. (2019)	Adults completed Author Recognition Task (classics, contemporary literary fiction, romance, fantasy, sci-fi, suspense, horror) and Big Five Inventory.	Sci-fi and fantasy positively correlated with openness.
Subbotsky, Hysted & Jones, 2010	In two studies, children watched film containing magical or non-magical content. Completed pre and post Torrance’s Thinking Creatively in Action and Movement Test scored for Fluency, Originality, and Imagination.	In both studies, magical film increased Fluency, Originality, and Imagination.
Black & Barnes (2021)	Students watched sci-fi film, a realistic film (crime drama or western) or nothing. Completed Transportation Scale and AUT Fluency.	For sci-fi films, greater transportation associated with increased Fluency.
Lin (2014)	High school students watched sci-fi film or taught problem-solving techniques. Completed Creativity Assessment Packet (CAP) and designed robotic cross-country vehicles.	Film increased CAP scores but not vehicle design creativity.
Shahghasemi & Khaleghipoor (2021)	Children interviewed about favourite animations and ideas for improving world. Informal analysis of watching fantasy vs other animations.	Fantasy animations associated with both fantastical and realistic ideas.
Black, Capps & Barnes (2018)	Adults completed Author Recognition Task (classics, contemporary literary, fantasy, horror, thriller, romance, sci-fi) and rated degree to which impossible events possible in future.	Sci-fi positively correlated with likelihood of impossible becoming possible.
**Virtual reality**		
Ritter et al. (2012)	Students experienced impossible VR, possible VR environment, or watched film of impossible VR. Completed AUT Flexibility.	Higher Flexibility after experiencing, but not watching, impossible events.
Moffat, Crombie & Shabalina (2017)	Students played Minecraft, a puzzle game, or a shooter game. Completed pre and post Torrance Tests of Creative Thinking for Originality, Flexibility or Fluency.	Minecraft didn’t increase creativity.
Blanco-Herrera, Gentile & Rokkum (2019)	Students played standard Minecraft, Minecraft plus instruction to be creative, a racing game or watched television. Completed RAT, AUT (Fluency and Originality) and draw an alien task (scored for creativity).	Standard Minecraft increased Fluency (compared to racing game) and drawing scores (compared to other conditions).
**Impossible dreams**		
Domino (1976)	High-school pupils classified as creative or non-creative *via* teacher’s ratings, AUT and RAT. Then kept a dream diary for 2 weeks.	Creative pupils’ dreams more impossible, bizarre, and uncanny.
Sladeczek & Domino (1985)	Adults from creative and non-creative professions kept a dream diary for a week.	Creative participants’ dreams more implausible, unrealistic, and bizarre.
Blagrove & Hartnell (2000)	Students completed Creative Personality Scale (CPS) and rated frequently of lucid dreaming.	Lucid dreamers obtained higher CPS scores than non-lucid dreamers.
Zink & Pietrowsky (2013)	Adults rated frequency of lucid dreaming and the Adjective Checklist (AC), scored for creativity.	Lucid dreamers obtained higher AC scores than non-lucid dreamers.
Tholey (1989)	Lucid dreamers asked dream characters to help complete various tasks, such as thinking of rhyming words or solving maths problems.	More successful with creative than logical tasks.
Stumbrys & Daniels (2010)	Lucid dreamers asked dream figures to help with creativity task (creation of new metaphors). Control group of non-lucid dreamers.	No difference in originality of answers.
**Anomalous experiences**		
Batey & Furnham (2008)	Students completed Oxford-Liverpool Inventory of Feelings and Experiences, self-ratings of creativity, CPS and The Biographical Inventory of Creative Behaviours (BICB).	Unusual Experiences positively correlated with self-ratings and BICB.
Schuldberg (2000)	Students completed the Perceptual Aberration–Magical Ideation scale (PAIMS), Barron–Welsh Revised Art Scale, How Do You Think, and creativity scales from the ACL.	PAIMS positively correlated with all creativity measures.
Thalbourne & Delin (1994)	Students, and adults diagnosed with depression and schizophrenia, completed Magical Ideation Scale and measure of creative personality based on the Torrance Creative Motivation Inventory.	Positive correlation between measures.
Kennedy & Kanthamani (1995)	In two studies, adults and students completed questionnaires asked about anomalous experiences and rated extent to which expressing creativity give their life meaning.	Measures positively correlated in both studies.
Ayers, Beaton & Hunt (1999)	Visual artists, actors and students completed The Hood Mysticism Scale and The Transpersonal Experience in Childhood and Adolescence Questionnaire.	Anomalous experiences higher in actors and visual artists.
Norlander, Kjellgren & Archer (2002–2003)	Students spent time in floatation tank or resting in a darkened room, and then completed Experienced Deviation from Normal State scale and produced essay rated for Originality.	Floatation tank resulted in more anomalous experiences and increased Originality.
Norlander, Bergman & Archer (1998)	Students either in floatation tank, relaxing on couch or sitting in chair reading magazines. Then completed version of AUT scored for Fluency and Originality.	Floatation tank produced higher Originality than reading group.
Braud (2012)	Qualitative study. Participants imagined reliving of exceptional or typical experiences, and described feelings.	Exceptional experiences involved broadening and creativity, typical experiences involved more ordinary functioning.

## Additional Information and Declarations

### Data Availability

The following information was supplied regarding data availability: This is a literature review and has no raw data.
